# Association of gender with cardiovascular and autonomic responses to central hypovolemia

**DOI:** 10.3389/fcvm.2023.1211774

**Published:** 2023-08-31

**Authors:** Vishwajeet Shankhwar, Janez Urevc, Bianca Steuber, Karin Schmid Zalaudek, Andrej Bergauer, Hanan Alsuwaidi, Stefan Du Plessis, Alawi Alsheikh-Ali, Catherine Kellett, Riad Bayoumi, Andrew Phillip Blaber, Nandu Goswami

**Affiliations:** ^1^ College of Medicine, Mohammed Bin Rashid University of Medicine and Health Sciences, Dubai, United Arab Emirates; ^2^ Faculty of Mechanical Engineering, University of Ljubljana, Ljubljana, Slovenia; ^3^ Division of Physiology, Otto Löwi Research Center of Vascular Biology, Immunity and Inflammation, Medical University of Graz, Graz, Austria; ^4^ Department of Surgery, General Hospital (LKH Südsteiermark), Wagna, Austria; ^5^ Department of Biomedical Physiology and Kinesiology, Simon Fraser University, Burnaby, BC, Canada; ^6^ Department of Integrative Medicine, Alma Mater Europea, Maribor, Slovenia

**Keywords:** gender, hemodynamics, autonomic, heart rate variability, lower body negative pressure, vascular, sex, sympathetic

## Abstract

**Introduction:**

Lower body negative pressure (LBNP) eliminates the impact of weight-bearing muscles on venous return, as well as the vestibular component of cardiovascular and autonomic responses. We evaluated the hemodynamic and autonomic responses to central hypovolemia, induced by LBNP in both males and females.

**Methodology:**

A total of 44 participants recruited in the study. However, 9 participants did not complete the study protocol. Data from the remaining 35 participants were analysed, 18 males (25.28 ± 3.61 years, 181.50 ± 7.43 cm height, 74.22 ± 9.16 kg weight) and 17 females (22.41 ± 2.73 years, 167.41 ± 6.29 cm height, 59.06 ± 6.91 kg weight). During the experimental protocol, participants underwent three phases, which included 30 min of supine rest, four 4 min intervals of stepwise increases in LBNP from −10 mmHg to −40 mmHg, and 5 min of supine recovery. Throughout the protocol, hemodynamic variables such as blood pressure, heart rate, stroke index, cardiac index, and total peripheral resistance index were continuously monitored. Autonomic variables were calculated from heart rate variability measures, using low and high-frequency spectra, as indicators of sympathetic and parasympathetic activity, respectively.

**Results:**

At rest, males exhibited higher systolic (118.56 ± 9.59 mmHg and 110.03 ± 10.88 mmHg, *p* < 0.05) and mean arterial (89.70 ± 6.86 and 82.65 ± 9.78, *p* < 0.05) blood pressure as compared to females. Different levels of LBNP altered hemodynamic variables in both males and females: heart rate [F(1,16) = 677.46, *p* < 0.001], [F(1,16) = 550.87, *p* < 0.001]; systolic blood pressures [F(1,14) = 3,186.77, *p* < 0.001], [F(1,17) = 1,345.61, *p* < 0.001]; diastolic blood pressure [F(1,16) = 1,669.458, *p* < 0.001], [F(1,16) = 1,127.656, *p* < 0.001]; mean arterial pressures [F(1,16) = 2,330.44, *p* < 0.001], [F(1,16) = 1,815.68, *p* < 0.001], respectively. The increment in heart rates during LBNP was significantly different between both males and females (*p* = 0.025). The low and high-frequency powers were significantly different for males and females (*p* = 0.002 and *p* = 0.001, respectively), with the females having a higher increase in low-frequency spectral power.

**Conclusions and future directions:**

Cardiovascular activity and autonomic function at rest are influenced by gender. During LBNP application, hemodynamic and autonomic responses differed between genders. These gender-based differences in responses during central hypovolemia could potentially be attributed to the lower sympathetic activity in females. With an increasing number of female crew members in space missions, it is important to understand the role sex-steroid hormones play in the regulation of cardiovascular and autonomic activity, at rest and during LBNP.

## 1. Introduction

Central hypovolemia refers to a condition in which blood volume within the central circulation decreases due to fluid shifts within the human body (1, 2). This usually occurs in circumstances where an individual remains in an upright position for a prolonged duration, leading to the accumulation of blood in the lower extremities and consequent impairment of blood flow to the heart and brain (3). Central hypovolemia triggers hypotensive activation of arterial and cardiopulmonary baroreflexes, which leads to a neurohumoral-mediated increase in heart rate and vasoconstriction to maintain blood pressure and blood flow to vital organs (4, 5). However, in some individuals, these compensatory responses are inadequate, and orthostatic intolerance develops (6). To assess a person's limit of cardiovascular stability during central hypovolemia, head up tilt and lower body negative pressure (LBNP) techniques are employed (7, 8). Both are widely used techniques that have shown that there are inter-individual differences in hemodynamic and autonomic responses during central hypovolemia, and that intra-individual responses are highly reproducible (9). LBNP is widely used as a countermeasure in spaceflight against the headward shifts of fluid in microgravity (10).

### 1.1. LBNP and gender differences

The magnitude of LBNP-induced cardiovascular and autonomic responses and pooling of blood in legs varies depending on the degree of negative pressure applied and the duration of exposure (2, 11–13). Several studies have investigated gender differences in the cardiovascular responses to LBNP and central hypovolemia (7, 14–17). A study by Thais et al. found that females had greater reductions in stroke volume and cardiac output in response to LBNP as compared to males, indicating a greater cardiovascular stress response in females (18). Another study by Pier et al. revealed gender-related differences in cardiovascular adjustments and adaptations to physical training (17). Several studies showed that females have a lower tolerance to central hypovolemic challenges compared to males (1, 7, 13, 18). In addition, differences in heart rate, sympathetic and adrenergic responses in males and females were noted during higher levels of hypovolemia. Overall, the studies appear to be inconsistent, as some studies found differences in cardiovascular responses and tolerance to low or high LBNP loading between males and females, whereas others did not. There are relatively few studies in the literature that have specifically investigated the effect of gender on LBNP responses.

The present study aimed to investigate how the level of LBNP (−10, −20, −30 and −40 mmHg) and gender (males and females) influence hemodynamic and autonomic systems. We hypothesized that there are (1): differences in resting hemodynamic and autonomic activity between genders; and (2) hemodynamic and autonomic responses to hypovolemia induced by LBNP will differ between genders.

A comprehensive understanding of potential gender differences in hemodynamic and autonomic nervous system responses to LBNP is critical to optimize interventions in a gender-specific manner. This is particularly important to advance our knowledge of the mechanisms underlying gender differences in cardiovascular health. By exploring these differences in greater detail, we may be able to identify new approaches to prevent and treat cardiovascular diseases tailored to the specific needs of males and females. Finally, with an increasing number of female crew members in manned space missions, it is important to understand the role that sex-steroid hormones play in regulating cardiovascular and autonomic activity, both at rest and during LBNP.

## 2. Materials and methods

The study was approved by Ethics committee of Medical University of Graz, Austria (Ref: EK 25-551 ex 12/13). All participants provided written consent prior to participation in the study.

### 2.1. Participants

This randomized cross-over study was conducted at the Division of Physiology, Medical University of Graz, Austria in 2022. The study enlisted young and healthy participants within the age range of 18–30 years, specifically excluding participants who had a history of smoking, alcoholism, psychological conditions, heart diseases, thrombosis, syncope or were pregnant. Participants were instructed to abstain from endurance sports or physical activity 48 h before the test and not to consume caffeine within 24 h before the experiment.

### 2.2. Study design and LBNP protocol

When the participants reached the laboratory, the study procedures were explained and then they were directed to lie down in a supine position. The experiment consisted of three phases: 30-minute rest period (baseline), 16-minute of various LBNP levels, and 5-minute recovery phase. To reduce the impact of circadian rhythms on the cardiovascular system, the study was conducted between 9.00 am and 1.00 pm (19). The investigations were carried out in a quiet, comfortable-lit room with temperature maintained at 23–25°C and humidity between 50% and 55%.

**Figure 1:**
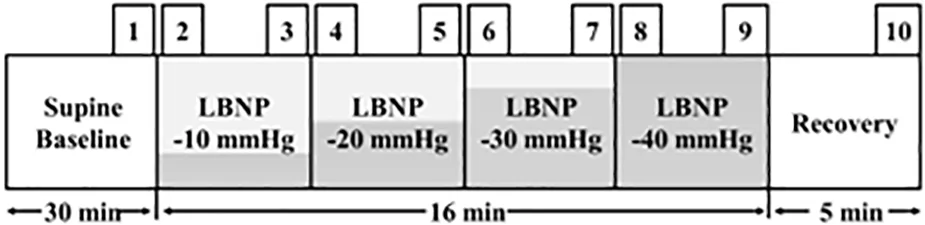
Study protocol and time points for data analysis. The study protocol shows that each LBNP level lasted 4-minute. The time points used for data analysis are also shown: each selected time point (1–10) had a duration of 10 s: the last 10 s of supine rest (baseline), at each LBNP level, the first 10 s (start) and the last 10 s, and the last 10 s of post LBNP application (recovery).

This study uses a graded LBNP protocol. After completion of 30-minute of supine rest (baseline), −10 mmHg LBNP was applied and then LBNP pressure was reduced by 10 mmHg every 4-minute, until −40 mmHg (total duration of LBNP is 16-minute, Figure 1). This protocol was chosen because −40 mmHg simulates fluid shifts induced standing upright (13), and almost all participants can tolerate this LBNP level. In cases where pre-syncopal signs or symptoms occurred, the LBNP application was immediately terminated. The criteria of pre-syncope were: (i) a drop in blood pressure below the systolic value of 80 mmHg or by a drop ≥20 mmHg/min, (ii) a drop in diastolic blood pressure ≥10 mmHg/min, (iii) a drop in heart rate ≥15 bpm, (iv) the occurrence of light-headedness, visual disturbances, nausea, clammy skin, or pallor skin, and/or (v) at the request of the participant (1, 15, 20, 21).

### 2.3. Data acquisition device

A Task Force® Monitor (TFM) 3040i from CNSystems Graz, Austria was used to continuously monitor physiological signals such as blood pressure, heart rate, and thoracic impedance measurements (22). Four electrodes were placed on the chest of the participants to obtain the electrocardiogram (ECG). The ECG was used to derive heart rate variability signals to assess autonomic responses (23, 24). Blood pressure was determined by a combination of right upper arm oscillometer and finger plethysmography.

### 2.4. Data analysis

Data from each participant were recorded during baseline, LBNP applications, and recovery (Figure 1). The study used specific time periods to analyze the data and observe the acute effect of LBNP application. Each selected time point (1–10) had a duration of 10 s: the last 10 s of supine rest (baseline), at each LBNP level, the first 10 s (start) and the last 10 s, and the last 10 s of post LBNP application (recovery). The recorded signals were processed and the following parameters were derived: heart rate, systolic blood pressure, diastolic blood pressure, mean arterial pressure, stroke index, cardiac index and total peripheral resistance index; and low-frequency-RRI (R-R intervals) and high-frequency-RRI band power derived from heart rate variability signal (25).

### 2.5. Statistical analysis

To ensure data privacy and security, the recorded data was stored on a password-protected laptop. OriginPro Lab 2022 was used for data evaluation, comparison, and understanding of changes in responses between male and female participants. Normality was checked by using the Kolmogorov–Smirnov test for the hemodynamic and autonomic data. Statistical differences between time points at baseline, different levels of LBNP, and recovery (Figure 1) were assessed using ANOVA and Bonferroni tests (*p* < 0.05). The baseline variables were compared between genders using student *t*-test (*p* < 0.05).

## 3. Results

A total of 44 adult participated in the study. However, 9 participants did not complete the study protocol due to fainting symptoms (3 males and 6 females). The results below are from the 35 participants, 18 males (25.28 ± 3.61 years, 181.50 ± 7.43 cm height, 74.22 ± 9.16 kg weight) and 17 females (22.41 ± 2.73 years, 167.41 ± 6.29 cm height, 59.06 ± 6.91 kg weight) who completed the protocol.

### 3.1. Resting cardiovascular and autonomic characteristics of males and females

Table 1 shows that males exhibited significantly higher systolic (118.56 ± 9.59 mmHg and 110.03 ± 10.88 mmHg, *p* < 0.05) and mean arterial (89.70 ± 6.86 mmHg and 82.65 ± 9.78 mmHg, *p* < 0.05) blood pressure as compared to females. In addition, mean diastolic blood pressure, stroke index, cardiac index, and total peripheral resistance were numerically higher in males, while heart rate was lower, but these differences were not statistically significant. In addition, males had higher low-frequency band power and lower high-frequency band power as compared to females, with the borderline significance of the differences (*p* = 0.053 and *p* = 0.054, respectively).

**Table 1:** Resting hemodynamic and heart rate variability parameters (which were used to calculate autonomic activity) in males and females.

Parameter	Male (*n* = 18)	Female (*n* = 17)	*p*-value
Heart rate (bpm)	62.74 ± 11.13	67.11 ± 12.28	0.142
Systolic blood pressure (mmHg)	118.56 ± 9.59	110.03 ± 10.88	0.010*
Diastolic blood pressure (mmHg)	73.59 ± 8.10	69.13 ± 9.99	0.081
Mean arterial pressure (mmHg)	89.70 ± 6.86	82.65 ± 9.78	0.021*
Stroke index (ml/m^2^)	59.80 ± 11.26	56.56 ± 11.27	0.204
Cardiac index (L/min/m^2^)	3.75 ± 0.91	3.68 ± 0.66	0.396
Total peripheral resistance index (dyn*sec*m^2^/cm^5^)	1,998.62 ± 580.87	1,852.96 ± 453.37	0.212
Low-frequency-RRI (n.u.)	54.20 ± 16.56	45.60 ± 15.56	0.053
High-frequency-RRI (n.u.)	45.61 ± 16.95	54.39 ± 15.57	0.054

Data are displayed as mean ± SD.

n.u., normalised unit.

* significant differences between males and females parameters (*p* < 0.05).

### 3.2. LBNP-induced heart rate and blood pressure responses in males and females

**Figure 2:**
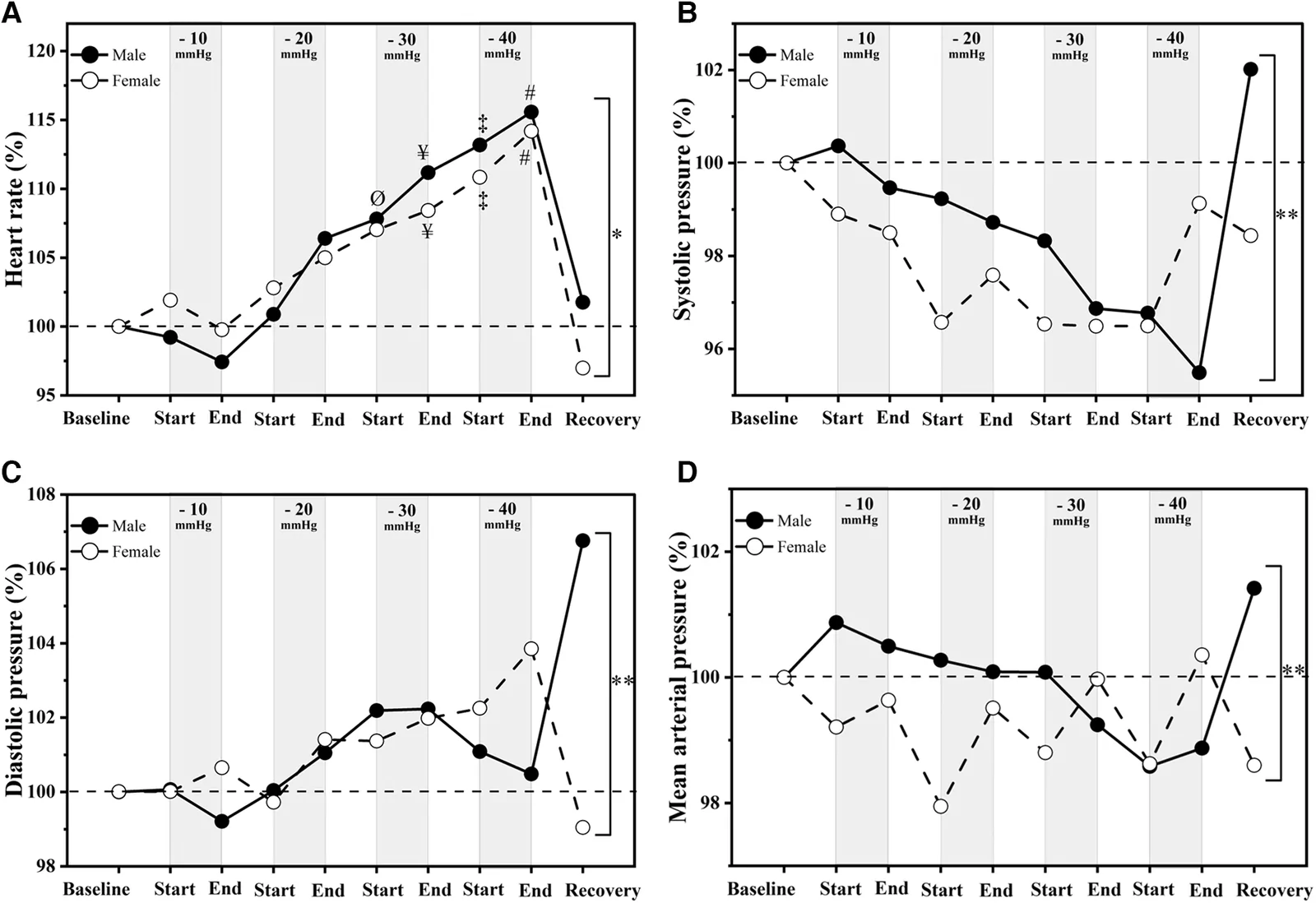
Hemodynamic responses to LBNP in (male (*n* = 18) vs. female (*n* = 17)). Data shown are relative changes (percentage) to the baseline: (**A**) Heart rate, (**B**) systolic pressure; (**C**) diastolic pressure and (**D**) mean arterial pressure. “Ø” significant differences between baseline and start of −30 mmHg; “¥” significant differences between baseline and end of −30 mmHg; “‡” significant differences between baseline and start of −40 mmHg; “#” significant differences between baseline and end of −40 mmHg (*p* < 0.05); “*” and “**” significant differences between males and females responses to LBNP (*p* < 0.05) and (*p* < 0.001), respectively.

The heart rate increased significantly at different levels of LBNP in both males [F(1,16) = 677.46, *p* < 0.001] and females [F(1,16) = 550.87, *p* < 0.001]. The increment in heart rates was significantly different between both genders (*p* = 0.025, Figure 2A). The systolic blood pressures at different levels of LBNP were significantly different in males [F(1,14) = 3,186.77, *p* < 0.001] and females [F(1,17) = 1,345.61, *p* < 0.001, Figure 2B]. Diastolic blood pressure responses were also significantly different in males [F(1,16) = 1,669.458, *p* < 0.001] and females [F(1,16) = 1,127.656, *p* < 0.001, Figure 2C]. Similarly, mean arterial pressures at different LBNP levels were significantly different for both males [F(1,16) = 2,330.44, *p* < 0.001] and females [F(1,16) = 1,815.68, *p* < 0.001, Figure 2D]. The systolic, diastolic and mean arterial blood pressure were significantly different between genders (*p* = 0.001, *p* = 0.001 and *p* = 0.001 respectively, Figure 2). It was observed that both males and females had a significant increase in heart rate at −30 mmHg and −40 mmHg (Figure 2A) in comparison to baseline. However, their heart rate returned to baseline within five minutes of the recovery phase. The effect of LBNP on diastolic (Figure 2B), systolic (Figure 2C) and mean arterial (Figure 2D) blood pressure at LBNP levels (−10, −20, −30 and −40 mmHg) was not statistically significant. However the responses were significantly different between genders.

### 3.3. LBNP-induced stroke, cardiac, and total peripheral resistance indices' responses in males and females

**Figure 3:**
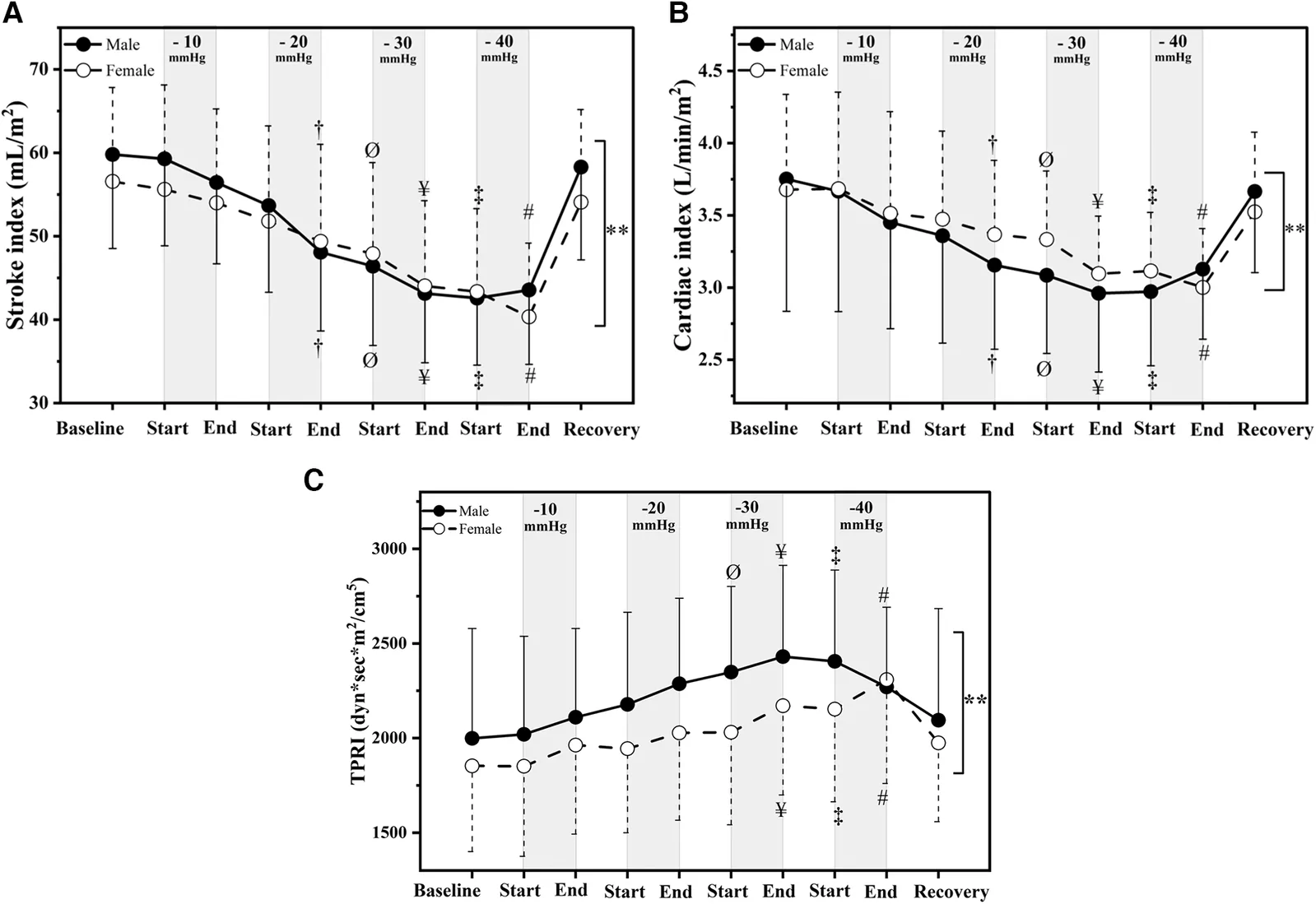
Cardiovascular compensatory responses to LBNP [male (*n* = 18) vs. female (*n* = 17)] in (**A**) stroke index, (**B**) cardiac index and (**C**) total peripheral resistance index (TPRI). Data are displayed as mean ± SD. “†” significant differences between baseline and end of −20 mmHg; “Ø” significant differences between baseline and start of −30 mmHg; “¥” significant differences between baseline and end of −30 mmHg; “‡” significant differences between baseline and start of −40 mmHg; “#” significant differences between baseline and end of −40 mmHg (*p* < 0.05). “**” significant differences between males and females responses to LBNP (*p* < 0.001) respectively.

The stroke index decreased significantly in males [F(1,16) = 620.95, *p* < 0.001] and females [F(1,15) = 367.331, *p* < 0.001], these reductions were significantly different between genders (*p* < 0.001, Figure 3A). Similarly, the cardiac index decreased significantly in males [F(1,15) = 496.76, *p* < 0.001] and females [F(1,15) = 716.196, *p* < 0.001], these decrements were also significantly different between genders (*p* < 0.001, Figure 3B). The reduction in stroke and cardiac indices were observed from −20 mmHg to −40 mmHg in females as compared to −20 mmHg to −30 mmHg in males (Figures 3A,B). During the recovery phase, both genders showed complete recovery of stroke index and cardiac index within 5-minute.

The total peripheral resistance index responses were significant in males [F(1,16) = 677.46, *p* < 0.001] and females [F(1,15) = 346.23, *p* < 0.001]. Notably, the responses differed significantly between genders (*p* = 0.006, Figure 3C). It increased in males from −10 mmHg to −30 mmHg. On other hand, it increased steadily in females until −40 mmHg. During the recovery phase, it returns completely to baseline in males and females (Figure 3C).

### 3.4. Effects of gender on LBNP-induced autonomic responses

**Figure 4:**
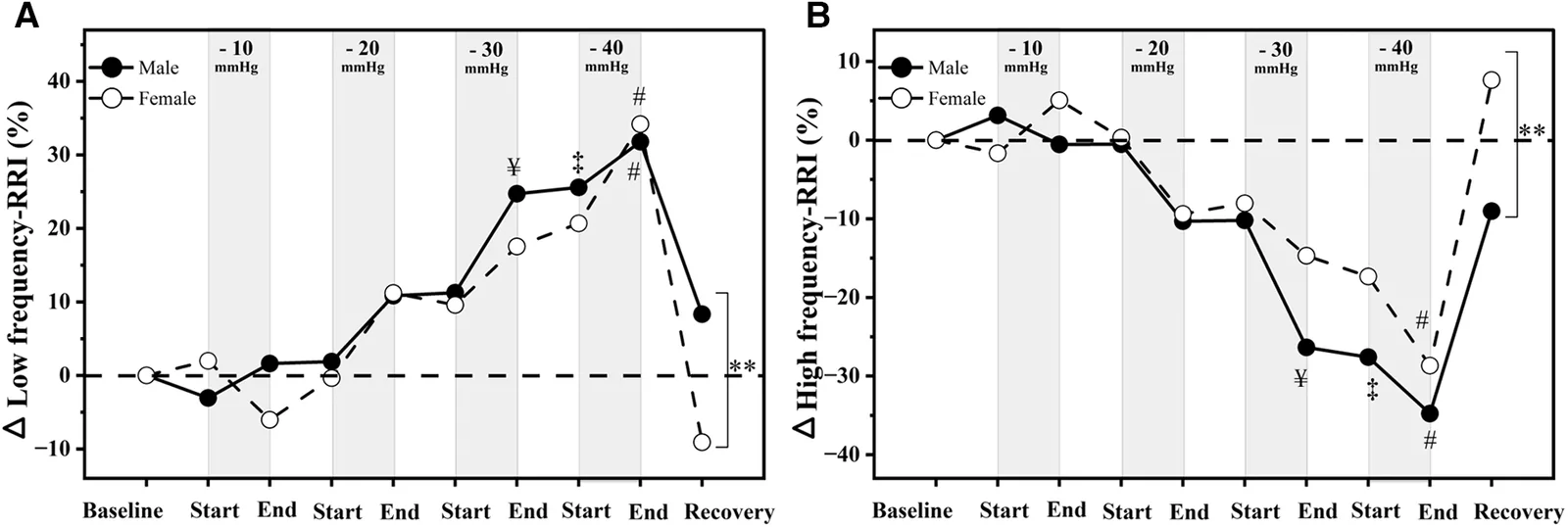
Relative changes in autonomic responses to LBNP [male (*n* = 18) vs. female (*n* = 17)] as compared to baseline. (**A**) low-frequency band power; (**B**) high-frequency band power.“¥” significant differences between baseline and end of −30 mmHg; “‡” significant differences between baseline and start of −40 mmHg; “#” significant differences between baseline and end of −40 mmHg (*p* < 0.05); “**” significant differences between males and females responses to LBNP (*p* < 0.001).

Low-frequency power increased significantly in males [F(1,15) = 220.46, *p* < 0.001] and females [F(1,16) = 200.217, *p* < 0.001, Table 2 and Figure 4A]. In contrast, high-frequency power decreased significantly in males [F(1,16) = 110.861, *p* < 0.001] and females [F(1,16) = 212.679, *p* < 0.001, Figure 4B] in response to LBNP. The variation in low and high-frequency powers were significantly different bewteeen genders (*p* = 0.002, *p* = 0.001 respectively, Figure 4), with the females having a higher increase in low-frequency spectral power. On the other hand, males had lower high-frequency spectral power at rest, which decreased at higher levels of LBNP. However, females experienced an even greater decrement in high-frequency power at higher levels of LBNP compared to males. During the recovery phase, low and high-frequency powers returned to baseline values in both males and females (Table 2 and Figure 4).

**Table 2:** Absolute values of autonomic responses during progressive LBNP in males and females.

Time-points		Low-frequency (n.u.)		High-frequency (n.u.)	
		Males (*n* = 18)	Females (*n* = 17)	Males (*n* = 18)	Females (*n* = 17)
1.	Baseline	54.39 ± 16.95	45.61 ± 15.58	45.61 ± 16.94	54.41 ± 15.58
2.	Start of −10 mmHg	53.13 ± 17.31	46.52 ± 16.61	46.87 ± 17.29	53.48 ± 16.62
3.	End of −10 mmHg	55.67 ± 19.06	42.85 ± 13.44	44.33 ± 19.05	57.15 ± 13.43
4.	Start of −20 mmHg	55.55 ± 18.51	45.45 ± 12.63	44.45 ± 18.49	54.55 ± 12.62
5.	End of −20 mmHg	59.31 ± 19.58	50.72 ± 17.56	40.69 ± 19.60	49.28 ± 17.55
6.	Start of −30 mmHg	59.93 ± 18.94	49.99 ± 17.84	40.07 ± 18.95	50.01 ± 17.85
7.	End of −30 mmHg	**67.93 **±** 15.36**^a^	53.61 ± 19.66	**32.07 **±** 15.37**^a^	46.39 ± 19.67
8.	Start of −40 mmHg	**68.15 **±** 16.36**^b^	55.05 ± 20.96	**31.85 **±** 16.35**^b^	44.95 ± 20.97
9.	End of −40 mmHg	**71.04 **±** 15.49**^c^	**61.20 **±** 18.91**^c^	**28.96 **±** 15.50**^c^	**38.80 **±** 18.89**^c^
10.	Recovery	58.29 ± 18.57	41.46 ± 14.85	41.71 ± 18.57	58.54 ± 14.85

Data are displayed as mean ± SD.

^a^ Significant differences between baseline and end of −30 mmHg.

^b^ Significant differences between baseline and start of −40 mmHg.

^c^ Significant differences between baseline and end of −40 mmHg; (*p* < 0.05).

## 4. Discussion

The purpose of this study was to investigate the changes in hemodynamic and autonomic parameters in healthy male and female participants at rest, as well as their responses to central hypovolemia induced by LBNP. The main results of this research indicate that there are gender-related differences in both resting hemodynamic and autonomic parameters, as well as in the hemodynamic and autonomic responses to LBNP application.

### 4.1. Resting cardiovascular and autonomic characteristics in males and females

Gender differences were seen in resting systolic, diastolic, mean arterial pressure, peripheral resistance, and low-frequency power of heart rate variability. These results indicate a gender-related difference in cardiovascular variables and autonomic function. These findings are consistent with previous research that has reported gender-related variations in blood pressure regulation, with males exhibiting higher levels of sympathetic nerve activity than females (26–29). The gender-specific differences in sympathetic tone could have contributed to the differences in hemodynamic and autonomic parameters between males and females at rest. Taken together, these findings suggest that there are important gender differences in cardiovascular parameters that should be considered in research and clinical practice, especially when males and females are included.

### 4.2. Gender and response to LBNP-induced central hypovolemia

At −10 mmHg LBNP, no significant changes in autonomic activity were observed in both males and females (Figure 4). However, females experienced a drop in systolic and mean arterial pressure, resulting in lower blood pressure compared to males. It is possible that a delay in sympathetic stimulation of the vasculature in females may account for this initial drop in blood pressure, while males did not experience a blood pressure decline in response to low-level LBNP due to their higher resting sympathetic tone. Moreover, the initial drop in blood pressure in females was followed by a subsequent decrease in stroke volume and cardiac output (−10 mmHg to −30 mmHg) (Figure 3). These reductions were similar in both genders, suggesting that the initial differences in blood pressure between males and females were not due to differences in cardiac function. A previous study also revealed similar results of reduction in stroke volume and a more significant decrease in cardiac output in females compared to males during graded LBNP (up to −50 mmHg) (30). The authors concluded that cardiovascular regulation and compensatory responses during central hypovolemia in females were largely dependent on cardiac output. Cheng et al. also reported that females had a weakened ability to respond to orthostatic blood pressure changes because of lower levels of sympathetic activity compared to males (31).

The present study indicates that during central hypovolemia heart rate, stroke and cardiac indices, and vascular resistance varied differently between genders. It shows that LBNP affects heart rate differently in males and females. These findings suggest that there are gender-specific differences in cardiovascular reactivity during central hypovolemia. These results are consistent with previous research conducted by Geelen et al. and Evans et al. who also reported gender-specific differences in cardiovascular responses during hypovolemia (20, 32). Geelen et al. observed that females had higher heart rate compared to males, which is in line with the results of the present study. Simonson and his colleagues investigated heart rate and blood pressure responses in seven high-tolerant males and eight low-tolerant males (33). They found that heart rate and blood pressure responses at −15 mmHg and −50 mmHg did not differentiate between high and low-tolerant orthostatic males. In the present study, we observed differences in heart rate and blood pressure responses between genders during LBNP.

Furthermore, our results indicate that as the pressure inside the LBNP chamber gradually changed from −20 to −40 mmHg, the low-frequency power (representing sympathetic activity) increased more in females, while the high-frequency power (representing parasympathetic activity) decreased as compared to males. Our results are in line with the studies conducted by Evans et al. who observed that females had lower sympathetic activity at rest and during hypovolemia resulting in blunted blood pressure and vascular resistance responses (20). The present study reveals that both resting and hypovolemic-induced changes in hemodynamic and autonomic parameters are influenced by gender. These findings are consistent with previous research that has reported anatomical and physiological differences between genders that affect cardiovascular and autonomic functions (1, 15, 17). Overall, the study highlights the potential of LBNP as a tool to evaluate gender differences in cardiovascular and autonomic responses.

## 5. Conclusions and future directions

Resting hemodynamic and autonomic parameters are influenced by gender. The effect of LBNP was different for both genders, which could potentially be attributed to the lower sympathetic activity in females. Sex-steroid hormones also play important roles also in the cardiovascular reactivity to the caudal-fluid shift induced by LBNP. Future studies should examine to what extent the menstrual phase influences resting hemodynamic and autonomic functions as well as the potential role different types of oral contraceptives play in cardiovascular and autonomic reactivity during central hypovolemia. With an increasing number of female crew members in manned space missions, it is important to understand the role sex-steroid hormones play in the regulation of cardiovascular and autonomic activity, at rest and during LBNP.

### 5.1. Limitations of the study

The study has a few limitations such as the lack of the absence of direct measurements of fluid shift volume in the lower extremities, which could provide more information about venous pooling during LBNP. Additionally, the exclusion of individuals with pre-existing conditions limits the generalization of the findings to unhealthy populations. Lastly, limitations that could be considered are the timing of the menstrual cycle and the use of contraceptives. These aspects can affect hormonal levels and cardiovascular function, potentially influencing the cardiovascular and autonomic responses to central hypovolemia. Future studies should consider incorporating them as a covariates to assess the potential impact on the observed outcomes.

## Data Availability

The raw data supporting the conclusions of this article will be made available by the authors, without undue reservation.
